# Occult blood flow patterns distal to an occluded artery in acute ischemic stroke

**DOI:** 10.1177/0271678X211044941

**Published:** 2021-09-22

**Authors:** Nerea Arrarte Terreros, Bettine G van Willigen, Wera S Niekolaas, Manon L Tolhuisen, Josje Brouwer, Jonathan M Coutinho, Ludo FM Beenen, Charles BLM Majoie, Ed van Bavel, Henk A Marquering

**Affiliations:** 1 Department of Biomedical Engineering and Physics, Amsterdam UMC, location AMC, Amsterdam, the Netherlands; 2 Department of Radiology and Nuclear Medicine, Amsterdam UMC, location AMC, Amsterdam, the Netherlands; 3 Cardiovascular Biomechanics, Eindhoven University of Technology, Eindhoven, the Netherlands; 4 Department of Neurology, Amsterdam UMC, location AMC, Amsterdam, the Netherlands

**Keywords:** Acute ischemic stroke, blood flow quantification, collateral circulation, dynamic CTA, thrombus permeability

## Abstract

Residual blood flow distal to an arterial occlusion in patients with acute ischemic stroke (AIS) is associated with favorable patient outcome. Both collateral flow and thrombus permeability may contribute to such residual flow. We propose a method for discriminating between these two mechanisms, based on determining the direction of flow in multiple branches distal to the occluding thrombus using dynamic Computed Tomography Angiography (dynamic CTA). We analyzed dynamic CTA data of 30 AIS patients and present patient-specific cases that identify typical blood flow patterns and velocities. We distinguished patterns with anterograde (N = 10), retrograde (N = 9), and both flow directions (N = 11), with a large variability in velocities for each flow pattern. The observed flow patterns reflect the interplay between permeability and collaterals. The presented method characterizes distal flow and provides a tool to study patient-specific distal tissue perfusion.

## Introduction

Acute ischemic stroke (AIS) occurs when a thrombus occludes an intracranial artery, severely restricting perfusion to brain tissue. The lack of blood supply leads to a rapidly growing oxygen debt, which can lead to irreversible neurological damage.

Despite the occlusion, blood may still flow in the arteries downstream of the thrombus. After stroke onset, the presence of distal flow depends on two factors: the permeability of the thrombus and the capacity of the cerebral collateral circulation. Evidence of thrombus permeability has been found in preclinical studies^1–3^ and clinical imaging studies.^4–6^ Thrombus permeability has been associated with favorable patient outcome and higher intravenous thrombolysis treatment success.^4–6^ The cerebral collateral circulation is a subsidiary vascular network that allows some remaining blood flow in case of an occlusion.^7–10^ Previous experimental research on rodents has presented the formation of collaterals after stroke onset, which influenced lesion volume and survival.^11,12^ Increased collateral flow has been related to favorable patient outcome.^13–16^

The level of remaining tissue perfusion (either due to permeability or collateral flow) is a major determinant of stroke outcome.^17,18^ The perfusion level discriminates between infarct, penumbra, and oligemic tissue.^19^ More remaining perfusion can sustain tissue at risk for a longer time, making intravenous or endovascular treatment a beneficial option for a longer time window. Still, the combined role of permeability and collaterals in distal perfusion is understudied.

Flow direction can reveal the source of distal tissue perfusion. Anterograde flow through the occlusion and downstream segments has been associated to permeable thrombi, while retrograde flow distal to the thrombus has been related to collateral flow coming from neighboring arterial branches.^20,21^ Flow direction has previously been assessed on dynamic Computed Tomography Angiography (dynamic CTA) data of AIS patients by manually placing markers on a single arterial segment immediately distal to the occlusion.^21,22^ However, this method neglects flow patterns in other distal branches and might therefore not completely characterize blood flow downstream of the thrombus. Important information on the interplay of thrombus permeability induced flow and collateral flow might be disregarded. Careful analysis of flow source is needed to better understand perfusion after stroke onset.

The aim of this study is to elucidate mechanisms of intracranial blood flow distal to the thrombus in AIS patients. We present a semi-automated method to characterize the direction and extent of blood flow in multiple branches distal to the thrombus using dynamic CTA data of AIS patients. We study patient-specific cases that portrayed typical flow patterns distal to the occlusion.

## Methods

### Data sets

We analyzed good quality thin-slice dynamic CTA data of 30 AIS patients who had a single occlusion in the middle cerebral artery (MCA). These patients were presented in our hospital between November 2017 and December 2018. The dynamic CTA data were acquired using a Siemens Somatom Force scanner (Siemens Healthineers, Forcheim, Germany) with a peak voltage of 70 kV and Hr36f convolutional kernel or a Siemens Somatom Definition AS scanners with a peak voltage of 80 kV and H20f convolutional kernel. The dynamic CTA scans were source images of CT perfusion (CTP) data, acquired in a routine clinical setting. Typical size of dynamic CTA images is $N_{x}\cdot N_{y}$ · $N_{z}=512\cdot 512\cdot 113$ (sagittal, coronal, axial, respectively), which were acquired at 30 different time points. Typical resolution of the data is Δx·Δy·Δz·Δt=0.44 mm·0.44 mm·1 mm·2s .

### Ethics

This study was conducted using observational data of patients from our hospital included in a prospective national multicenter registry (MR CLEAN Registry).^23^ This registry was approved by the Erasmus University Medical Center Central Ethics Committee, which served as the central review board for all participating centers. The requirement for written informed consent was waived, but all patients or legal representatives were provided with information on the registry orally and in writing, and had the opportunity to withdraw consent to use their data via an opt-out form, conforming to the European Union General Data Protection Regulation. The registry procedures followed were all in accordance with the Declaration of Helsinki, as amended by the World Medical Association General Assembly in October 2008.

### Vessel segmentation

**Figure 1.**
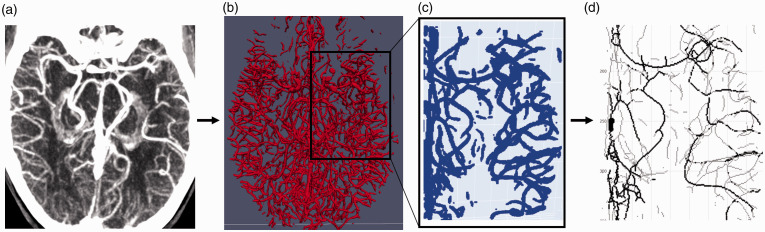
Illustration of the pre-processing steps: (a) creation of the temporal maximum intensity projection, (b) segmentation of the intracranial vessels, (c) definition of the region of interest (ROI) that encapsulates the occlusion, and (d) skeletonizing of the defined ROI.

In order to quantify blood (contrast) flow direction distal to the occluded MCA, we developed a pipeline to determine the vessel centerlines. We pre-processed each image set by first stripping the skull and registering every timeframe to the first timeframe using Elastix’ rigid registration.^24^ The data were filtered using a bilateral filter.^25^ For each data set, we computed a temporal maximum intensity projection (t-MIP) (Figure 1(a)). From this t-MIP, we segmented all vessels of the anterior circulation using a U-NET model provided by Nico.lab,^26^ similar to a previous study^27^ (Figure 1(b)). We subsequently defined a region of interest (ROI) that contained the downstream territory of the occluded MCA segment using ITK-snap^28^ (Figure 1(c)). We skeletonized the vessels of interest by iterative morphological thinning of a binarized image, while ensuring connectivity and geometry (Python’s skeleton library). The skeletons of the vessels correspond to the vessel centerlines (Figure 1(d)).

### Vessel selection

**Figure 2.**
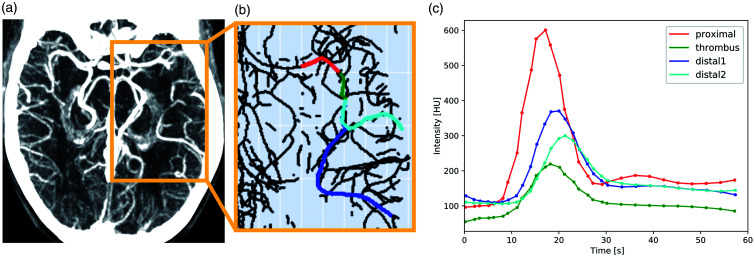
(a) Maximum intensity projection of a dynamic CTA scan with a left middle cerebral artery occlusion. (b) Example of vessel centerlines. The vascular paths proximal to the thrombus, within the thrombus, and distal to the thrombus are colored in red, green, and blue, respectively. (c) Typical raw time attenuation curves (TAC) of contrast intensity [HU]. Each TAC corresponds to the dynamic attenuation within a single voxel. Colors as in (b), with two distal paths.

We developed a graphical user interface (GUI) using JavaScript to assist the computation of the flow direction in multiple branches distal to the occlusion. This GUI allows the selection of the arterial branches of interest and the placement of reference markers indicating the proximal branch, thrombus branch, and distal branches. In case of a bifurcating thrombus, the branch with a longer thrombus was selected. The complete path along the artery centerline was subsequently computed based on the placed reference markers. These paths were determined using a path-finding algorithm that computes the minimum-cost path based on the intensities in the t-MIP: high intensities yield a low cost; low intensities yield a high cost. An example of the extracted paths can be found in (Figure 2(a) and (b)).

The manual selection of the arterial branches was performed by multiple trained observers, cognizant of the occlusion location reported by the attending neuroradiologist. These selections were discussed a posteriori and adjusted if necessary to capture the optimal topology.

### Blood flow direction

For each voxel along the centerline of the branches of interest, the contrast intensity over time was determined and displayed as time attenuation curves (TACs) (Figure 2(c)). These raw TACs were interpolated and filtered using a Butterworth low-pass filter to remove high-frequency noise in time.

The arrival time of contrast differed per voxel. The difference in arrival time between locations (time delay) was computed using a cross-correlation between the TACs of each marker and the most proximally located marker. We discarded TACs with a maximum intensity less than 5% of the maximum intensity of the most proximal TAC.

To determine the direction of the flow, the computed time delay was analyzed as a function of the distance from the most proximal marker.^21^ If the time delay increased with the distance along the vessel, the contrast was assumed to be moving distal, reflecting anterograde flow. Conversely, if the time delay decreased with the distance, the contrast was considered to be moving towards the thrombus, reflecting retrograde flow.

### Blood velocity

The blood velocity along the distal branches was estimated by fitting a linear regression along the time delay over distance. The inverse of the slope corresponds with the average velocity. If the distal branches had bifurcations, a linear regression was fitted to each segment.

## Results

Baseline characteristics of the 30 patients can be found in Supplementary Material, Appendix A. We found a large variety of vessel topologies distal to the occlusion, which included vessel bifurcations and trifurcations. To facilitate the analysis of these distal branches, we distinguished between mother and daughter segments (see Supplemental Material, Appendix B, Figure 1). Based on the downstream flow directions of the daughter segments, we grouped the patients (see Supplementary Material, Appendix B):

- Pattern I: anterograde distal flow (N = 10/30 patients).
- Pattern II: retrograde distal flow (N = 9/30 patients).
- Pattern III: both anterograde and retrograde distal flow (N = 11/30 patients).

A summary of the blood flow patterns for all 30 patients can be found in Supplementary Material, Appendix B. Below we present four patient-specific cases that illustrate these distal flow patterns.

### Pattern I: Anterograde distal flow

**Figure 3.**
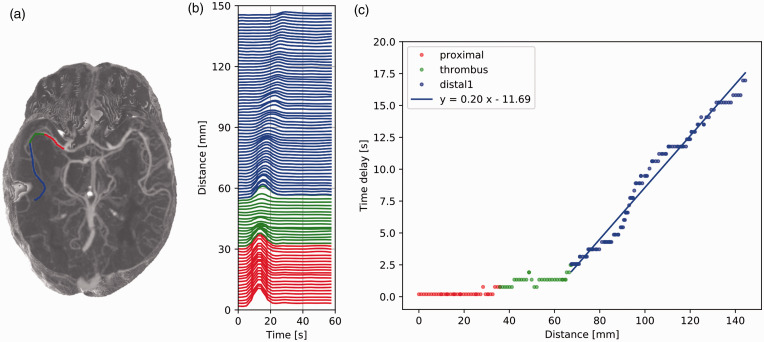
Patient I-a: slow anterograde flow. (a) Maximum intensity projection of the dynamic CTA scan of patient I-a with a right middle cerebral artery occlusion. Vessel segments proximal to the thrombus, within the thrombus, and distal to the thrombus are colored in red, green, and blue, respectively. (b) Time attenuation curves (TACs) along the vessel segments, colored as in (a). The Y axis scale denotes the position of the voxel from where the TACs were extracted. (c) Time delay [s] as a function of the distance [mm]. The results of the cross-correlation (dots) are fitted with a linear regression for the distal branch (blue line). The positive slope indicates anterograde flow and its inverse corresponds to the average blood velocity: 5 mm/s.

**Figure 4.**
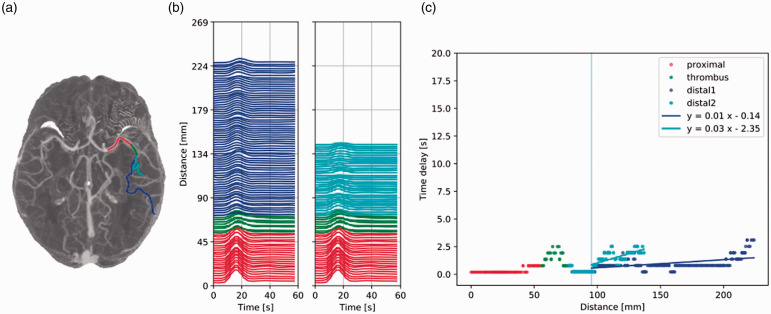
Patient I-b: fast anterograde flow. (a) Maximum intensity projection of the dynamic CTA scan of patient I-b with a left middle cerebral artery occlusion. Vessel segments proximal to the thrombus, within the thrombus, and distal to the thrombus are colored in red, green, and (light and dark) blue, respectively. (b) Time attenuation curves (TACs) along the vessel segments, colored as in (a). The Y axis scale denotes the position of the voxel from where the TACs were extracted. (c) Time delay [s] as a function of the distance [mm]. The results of the cross-correlation (dots) are fitted with a linear regression for each distal branch. The positive slope indicates anterograde flow and its inverse gives an estimation of the average blood velocity. Distal to the thrombus and up to the bifurcation (vertical line), the time delay is zero (meaning that contrast appearance could not be detected due to the limited time resolution of the data). Further distally, flow is anterograde and the velocities are 100 mm/s and 33 mm/s for distal1 and distal2 (dark and light blue lines), respectively.

Patient I-a and I-b showed anterograde flow proximal to, within and distal to the occlusion. One distal branch was identified for patient I-a, while two branches were identified for patient I-b. Patient I-a presented larger time delays and larger spreading of the distal TACs compared to patient I-b. The anterograde distal flow velocity of patient I-a was much lower than that of patient I-b, with average velocities of 5 mm/s vs. 100 mm/s and 33 mm/s, respectively (Figures 3 and 4).

### Pattern II: Retrograde distal flow

**Figure 5.**
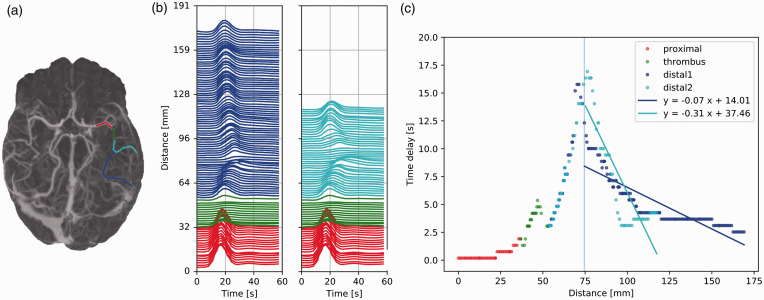
Patient II-a: retrograde flow. (a) Maximum intensity projection of the dynamic CTA scan of patient II-a with a left middle cerebral artery occlusion. Vessel segments proximal to the thrombus, within the thrombus and distal to the thrombus in red, green, and (light and dark) blue, respectively. (b) Time attenuation curves (TACs) along the vessel segments, colored as in (a). The Y axis scale denotes the position of the voxel from where the TACs were extracted. (c) Time delay [s] as a function of the distance [mm]. The positive and negative slopes indicate anterograde and retrograde flow, respectively. Up to the vessel bifurcation (vertical line), flow in anterograde. Distal to the bifurcation, flow is retrograde and the velocities are −14 mm/s and −3 mm/s for distal1 and distal2 (dark and light blue lines), respectively.

For patient II-a, flow direction proximal to, within and immediately distal to the occlusion was anterograde. However, further distal, after the vessel bifurcation, retrograde flow was observed. Retrograde flow close to the cortical territory was fast, and it slowed down near the vessel bifurcation. Anterograde flow and retrograde flow merged at the vessel bifurcation. In-thrombus TACs were more dispersed and had lower intensities than proximal TACs. Distal TACs showed a similar behavior as thrombus TACs in the case of anterograde flow. Distal TACs were less dispersed and had higher intensities than thrombus TACs when retrograde flow was found. Distal to the bifurcation, the velocities were −14 mm/s and −3 mm/s for the distal arteries (Figure 5).

### Pattern III: Both anterograde and retrograde distal flow

**Figure 6.**
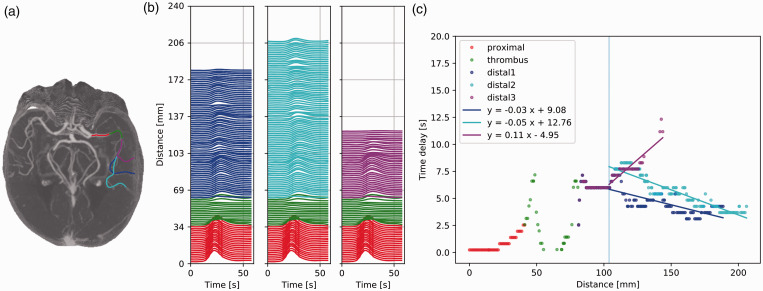
Patient III-a: both anterograde and retrograde flow. (a) Maximum intensity projection of the dynamic CTA scan of patient III-a with a left middle cerebral artery occlusion. The vessel segment proximal to the thrombus is colored in red, within the thrombus in green, and distal to the thrombus in (light and dark) blue and magenta. (b) Time attenuation curves (TACs) along the vessel segments, colored as in (a). The Y axis scale denotes the position of the voxel from where the TACs were extracted. (c) Time delays [s] as a function of the distance [mm]. The positive and negative slopes indicate anterograde and retrograde flow, respectively. Distal to the thrombus and up to the vessel trifurcation (vertical line), the time delay is zero (meaning that contrast appearance could not be detected due to the limited time resolution of the data). Further distally, the average velocities are −33 mm/s, −20 mm/s, and 9 mm/s for distal1, distal2, and distal3 (dark blue, light blue, and magenta lines), respectively.

Patient III-a showed anterograde flow proximal to, within and immediately distal to the occlusion. Further distal, after the vessel trifurcation, flow was retrograde in two branches and anterograde in one branch. TACs of the anterograde branch were more dispersed and had lower intensities than TACs of the retrograde branch. Retrograde flow was faster than anterograde flow. The average velocities were −33 mm/s, −20 mm/s, and 9 mm/s (Figure 6).

## Discussion

In this study we presented a method to characterize blood flow in multiple branches distal to a middle cerebral artery occlusion and we have illustrated that these flow patterns vary greatly in patients with an AIS. We have identified flow patterns with anterograde flow, retrograde flow, and both flow directions.

Recent studies on rodent stroke models have also reported findings of retrograde flow coming from collaterals^11,12,29,30^ and flow patterns with both anterograde and retrograde flow distal to the occlusion.^31^ Other studies have visually assessed blood flow direction on dynamic CTA data of AIS patients.^20,32^ Visual inspection of flow is mostly limited to the distinction of patients with predominant anterograde or retrograde flow and hinders the identification of more complex blood flow patterns. Flow direction has previously been quantified in a single arterial branch, where flow patterns were dichotomized into anterograde and retrograde flow.^21^ Measuring flow in a single arterial segment immediately distal to the occlusion leads to an oversimplification of the flow patterns. As shown in the cases of retrograde and both flow directions (pattern II and III), only considering a short segment distal to the occlusion can neglect the retrograde collateral flow, and therefore, assume that tissue further distally from the occlusion is not perfused (fast enough). To better understand and quantify tissue perfusion after stroke onset, flow patterns in multiple branches up to the cortex should be studied and quantified.

The presented variation in blood flow patterns manifests the different interplays between the permeability of the thrombus and the performance of collaterals. The flow provided by each of these mechanisms is delimited by the mechanism itself: the physical properties of the thrombus define the permeability, and the inherent collateral angioarchitecture of the patient is a delimiting factor of the maximum collateral performance.^33,34^ At the same time, the flow resulting from these mechanisms depends on the performance of the other: thrombus permeability affects the pressure distal to the occlusion, which is driving the collateral flow, and vice versa. For clarity, we present flow scenarios depending on the balance of collaterals and permeability in Figure 7, and its relation to the found flow patterns:

**Figure:**
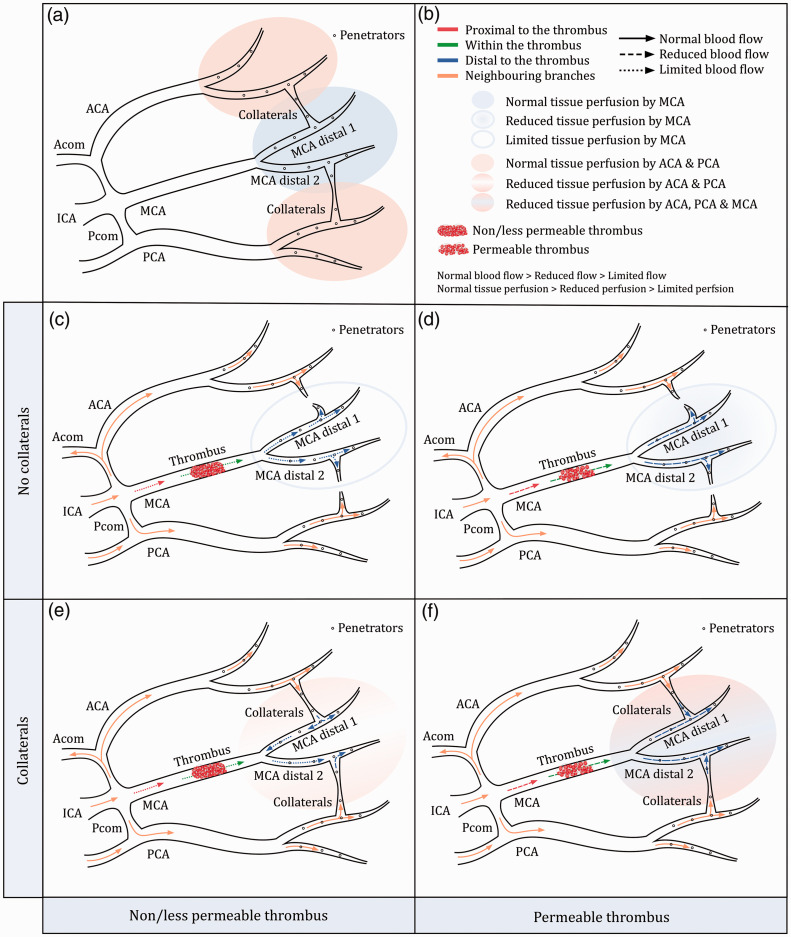
Figure 7. (a) Schematic drawings of the internal carotid artery (ICA), anterior cerebral artery (ACA), middle cerebral artery (MCA), posterior cerebral artery (PCA), anterior communicating artery (Acom), posterior communicating artery (Pcom), collateral circulation and penetrating arteries (penetrators). The schematic drawing is an oversimplification of the real anatomical structures. The MCA bifurcation does not merely represent the M1-M2 bifurcation, but rather any vessel bifurcation distal to the occlusion. The major leptomeningeal collaterals are in reality end-to-end anastomoses. (b) Figure legend. Flow direction is indicated by the direction of the arrow. Flows proximal to the thrombus, within the thrombus, and distal to the thrombus are colored in red, green, and blue, respectively. Blood flow through neighboring arteries (ICA, ACA, PCA, Pcom, and Acom) is colored in orange. We distinguish between (quasi-)normal, reduced, and limited blood flow, and between (quasi-)normal, reduced, and limited tissue perfusion. In both cases, normal > reduced > limited. Perfusion coming from the MCA is denoted in blue. Perfusion coming from the neighboring ACA and PCA is denoted in orange. Thrombi are classified into permeable and non/less permeable thrombi. (c) Non/less permeable thrombus with no collaterals. The absence of collaterals is due to a poor collateral angioarchitecture or poor collateral performance. The perfusion of the MCA territory is only dependent on the limited residual anterograde flow coming from the thrombus. (d) Permeable thrombus with no collaterals. The absence of collaterals is due to a poor collateral angioarchitecture or poor collateral performance. Tissue perfusion of the MCA territory relies on the residual anterograde flow coming from the thrombus. (e) Non/less permeable thrombus with collaterals. Tissue perfusion of the MCA territory is mainly dependent on the collateral flow coming from the ACA and PCA. The limited flow coming from the thrombus may also contribute to tissue perfusion. This situation allows flow patterns with both anterograde and retrograde flow. (f) Permeable thrombus with collaterals. The perfusion of the MCA territory relies on both the residual anterograde flow coming from the thrombus and the collateral flow coming from the ACA and PCA.

- Pattern I. Exclusive anterograde flow indicates cases where distal perfusion primarily depends on the permeability of the thrombus. Fast anterograde flow is related with a permeable thrombus (or incomplete occlusion by the thrombus) that allows residual flow (Figure 7(d) and (f)). Slow anterograde flow is due to a less permeable thrombus (Figure 7(c)). Collaterals can or cannot contribute to the distal anterograde flow. The lack of collateral flow indicates that either due to the (high) thrombus permeability the pressure drop is not large enough to drive flow retrogradely towards the occlusion, or the extent of the collateral circulation is not sufficient to provide blood to the target downstream territory.
- Pattern II. The presence of retrograde flow downstream of the occlusion is due to the performance of the collateral circulation (Figure 7(e)). In this case, the thrombus may still allow residual anterograde flow through the occlusion. The created pressure gradient drives collateral flow retrogradely towards the thrombus. This type of patient might be similar to a patient with exclusive slow anterograde flow (Figure 7(c)) but a good collateral architecture (Figure 7(e)).
- Pattern III. The presence of both anterograde and retrograde flow is due to the combined effect of permeability and collaterals, or a consequence of collateral flow going retrogradely and then anterogradely through a side branch (Figure 7(e)).

We reported flow velocities in the range of 1–100 mm/s distal to the occlusion. Flow velocity differences between branches may occur due to the flow division at nodes as well as differences in vascular diameter (since flow equals velocity times cross-sectional area). There is a paucity of data on variations in flow velocity between branches under normal (or minimally interrupted) conditions. Distal to an occlusion, the range of 1–100 mm/s seems possible, given that under normal conditions, blood velocity in the MCA has been reported to be 400–600 mm/s.^35^

In the context of personalized medicine, having access to a method to quantify patient-specific blood flow characteristics opens the possibility to better understand and determine distal tissue perfusion. It is expected that distal perfusion moderates the pace of infarct progression.^36^ For the same time point after stroke onset, a patient with a good collateral system feeding the hypoxic brain territory might have a smaller infarct core and larger penumbra than a patient with poor collaterals.^14,16,18,19,37,38^ A permeable thrombus may allow residual anterograde flow downstream of the occlusion. A patient with anterograde flow through the thrombus might benefit from thrombolysis even after the recommended treatment window.^5^

Thrombus permeability is influenced by many parameters such as thrombus length, void fraction and/or histology.^22,39–41^ The permeability of the thrombus affects the pressure drop over the occluded vessel, and this pressure drop determines the direction of the flow distally. A highly impermeable thrombus can cause a large pressure drop over the occlusion, which can drive retrograde, collateral-related flow towards the thrombus. This pressure drop is partly influenced by the mean arterial pressure of the patient. Increased arterial pressure has also been related to increased collateral flow.^42,43^ For a given collateral network and thrombus permeability, we could expect that both collateral flow and thrombus flow increase to a similar extent at higher central blood pressures, without a fundamental change in the flow patterns.

Future research could focus on the association of dynamic CTA-based flow measurements with CTP perfusion analyses. CTP provides information on perfusion level of the affected brain hemisphere, but it does not provide any information on the source of this perfusion. The combination of both flow direction and perfusion measurements may contribute to the understanding of perfusion mechanism after stroke onset.

## Limitations

This study has some limitations. The temporal resolution of the dynamic CTA scans was 2 s. This leads to around a 1 s error in the cross-correlation-based time delay estimate. We could therefore not resolve the time differences in vessel segments with fast flow. The computation of the velocity was based on a linear fit of the data, which is an oversimplification of the observed complex local velocities. However, it does illustrate the observed variability (e.g. the velocity difference observed in patient I-a vs. patient I-b). Contrast flow can be affected by patient factors such as the anatomy of the Circle of Willis, the presence of proximal stenosis in the carotids, or the cardiac output. In-thrombus TACs were often disperse, which could affect the computed time delay.

We analyzed flow velocity and direction in a few major branches distal to the thrombus. As a general rule, the branches were followed up to the cortex, but this was not always feasible. The observed flow patterns are far from trivial. Clearly, there are many side branches and further bifurcations distal to the identified “daughter segments” that affect the flow patterns. An apparent violation of flow conservation in our analyzed branches, therefore, can be caused by small side branches that were not included by the observer or lost in the segmentation process.

The validation of the method and measurements using, for example, other imaging modalities is difficult. To some extent, flow direction can be judged in digital subtraction angiography (DSA). However, a quantitative and per-branch flow analysis on DSA seems not feasible, considering the 2 D nature of such imaging. On the other hand, CTP data provides information on the level but not on the source of tissue perfusion, and thereby is difficult to link to the flow directions in the proximal segments. In addition, the translation from perfusion to velocity requires assumptions on among others perfusion territory and diameter of the analyzed segments. Therefore, the presented method should be considered a proof of concept showing the complexity and variability of flow patterns distal to the thrombus rather than a validated flow assessment methodology.

The anterograde in-thrombus flows found in this study represent the ability of nanometer-sized contrast to pass through the thrombus, and not that of whole blood, containing the micrometer-sized red blood cells (RBC) that provide tissue oxygenation. We could not establish whether the permeable thrombi allowed the RBC to pass. Nevertheless, the presence of anterograde in-thrombus flow might still be beneficial for thrombolysis.

The use of this method in large clinical trials requires automated thrombus detection and branch selection in order to save analysis time and observer dependency.

Finally, the current study did not include enough patients to statistically address (clinical) differences between the presented flow patterns, such as differences in Alberta stroke programme early CT score (ASPECTS) or baseline National Institute of Health Stroke scale (NIHSS), which may have some influence of intracranial flow patterns.

## Conclusion

We have shown that there is a large variety of flow patterns distal to the occluding thrombus in patients with an acute ischemic stroke. We have identified anterograde and retrograde flow patterns in multiple middle cerebral artery branches distal to the occlusion. This characterization can help understanding the different roles of thrombus permeability and collateral circulation and opens the possibility to study patient-specific distal tissue perfusion.
